# Indirect Mode of Suture of the Nerves

**Published:** 1918-12

**Authors:** 


***Indirect Mode of Suture of the Nerves.*** By M. J. NAGEOTTE. *Bulletin de la Société de Chirurgie de Paris.* June 18, 1918.

A certain number of tests have been undertaken by the author to find out the best method of treating injured nerves when the loss of nervous substance is not so great as to render the coaptation of both extremities impossible.

An injured nerve never heals by first intention. The details of the repairing process may be observed when a nerve graft is set in between the extremities of the injured nerve. A small bulb appears on the superior end, the neuroma; from this grows soon a thin tractus extending towards the other extremity. The same change takes place at the lower end, but with much less vigor; no neuroma develops there, but a glioma, i. e., a nervous neoplasm produced by the growth of the sheaths of Schwann. Both extremities of the tractus unite and form a sort of passage for the regenerated fibers.

Supposing now a direct suture of both ends of the injured nerve should be performed. The activity of the cells, which in the former case induces them to form the bulbs, exists just the same. A small fibrous bulb develops in which a tumor representing altogether the neuroma, glioma, and tractus may be found.

Does this lead to some disturbances in the repairing of the nerve? The author experimented this by using a heterogenous nerve graft taken from a foetus of calf and kept in alcohol.

Experiments were made on 6 dogs, and the sciatic nerves of these animals were cut. On one side both extremities of the nerve were directly sutured; on the other a 5mm. long nerve graft was introduced between both ends of the nerve. In two cases the whole sciatic nerve was cut through; in 4, only the infernal popliteal sciatic.

The author obtained the following results:

Some weeks after the operation, three dogs presented severe trophic disturbances, together with a considerable muscular atrophy in the leg which had undergone the direct suture, whereas the other one was perfectly sound.

In the three other cases, results were the same in both legs, or only slightly better on the side of the graft.

The author considers, therefore, that the direct suture of a nerve may be followed by severe trophic disturbances in the limbs, whereas the indirect suture performed with a dead nerve graft proves to have better results.

Histological examination shows that the number and size of the regenerated fibers are rather larger in the case of the direct suture. This proves simply that, as regards the functional repair, the number of regenerated fibers is less important than the way in which this regeneration occurs.

The direct suture was performed with 2 or 3 silk threads. The grafts were killed by immersion in alcohol at 50 per cent., and kept in sealed tubes, where they might be retained for a few weeks at least. It is quite unnecessary to cut the graft from a human nerve; the fact of using a heterogeneous graft is of no importance at all.

